# Interfacial Engineering Facilitates Real-Time Detection of Dual Hazardous Gases at ppb Levels via Single-Step Hydrothermal Nanoarchitectonics of Self-Assembled PbSnS/SnO_2_ Heterostructures

**DOI:** 10.1021/acssensors.4c03215

**Published:** 2025-01-30

**Authors:** Utkarsh Kumar, Yu-Wen Yeh, Zu-Yin Deng, Wen-Min Huang, Chiu-Hsien Wu

**Affiliations:** † Department of Physics, National Chung Hsing University, Taichung 402, Taiwan; ‡ Institute of Nanoscience, National Chung Hsing University, Taichung 402, Taiwan

**Keywords:** ternary metal sulfide, metal oxides, heterostructures, gas adsorption, DFT

## Abstract

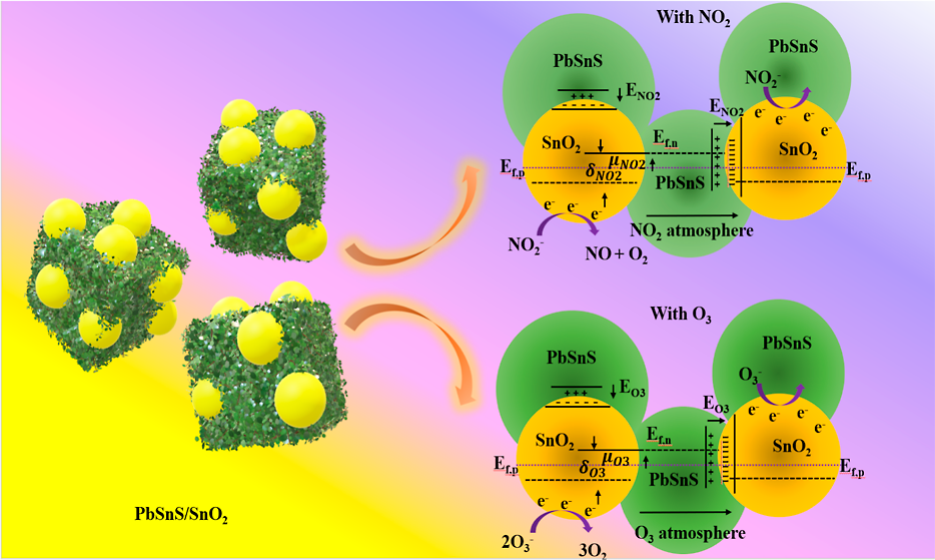

Next-generation real-time gas sensors are crucial for detecting multiple gases simultaneously with high sensitivity and selectivity. In this study, ternary metal sulfide (PbSnS)-incorporated metal oxide (SnO_2_) heterostructures were synthesized via a one-step hydrothermal method. Characterizations such as X-ray diffraction, high-resolution transmission electron microscopy, and X-ray photoelectron spectroscopy confirmed the successful formation of PbSnS/SnO_2_ heterostructures. Subsequently, thin films based on PbSnS/SnO_2_ heterostructures were fabricated and employed for the detection of real-time dual hazardous oxidizing gases at room temperature. The sensor response for NO_2_ gas was found to be 1.04 at 25 parts per billion (ppb) with a limit of detection (LOD) of 18.17 ppb, while for O_3_ gas, the sensor response was 1.03 at 15 ppb with an LOD of 7.34 ppb. Moreover, high selectivity for detecting two oxidizing gases in real time by using differential analysis of the gas sensing curve has been reported. Furthermore, density functional theory calculations corroborated the sensing mechanism, elucidating that the Pb atom in PbSnS/SnO_2_ is primarily responsible for the adsorption of NO_2_ gas, whereas SnO_2_ in PbSnS/SnO_2_ is responsible for the adsorption of O_3_ gas. These findings demonstrate the potential of PbSnS/SnO_2_ heterostructures for advanced gas sensing applications, offering insights into their fundamental sensing mechanisms.

Gas sensors are integral components in various applications, playing an essential role in the detection and quantification of specific gases within the environment.^1,2^ Their indispensability stems from diverse factors, and their utilization spans a broad spectrum. Here are outstanding technical aspects underscoring the necessity and applications of gas sensors. Gas sensors assume a critical role in industrial surroundings where the potential exists for exposure to noxious or combustible gases.^3,4^ They contribute to the establishment of a secure working environment by discerning and promptly signaling the presence of hazardous gases. Gas sensors serve as instrumental tools in the continuous surveillance of air quality and are applicable in urban locales, industrial areas, and confined spaces. These sensors adeptly identify pollutants, including carbon monoxide, sulfur dioxide, nitrogen dioxide, and particulate matter, facilitating the evaluation and amelioration of environmental repercussions.^3,5−7^

Within healthcare sectors, gas sensors are strategically deployed to monitor the concentration levels of specific gases such as oxygen and carbon dioxide in patient surroundings, notably in healthcare facilities such as hospitals and clinics. The technical workings of gas sensors include sophisticated methodologies for the precise detection of gas concentrations. Utilizing diverse sensing technologies, such as electrochemical, semiconductor, and optical methods, these sensors provide accurate readings and timely alerts in response to the presence of target gases. In industrial contexts, gas sensors are often integrated into comprehensive safety systems, employing advanced algorithms for real-time data analysis and decision-making. Moreover, in environmental monitoring, gas sensors influence advanced calibration techniques and data fusion algorithms to ensure accurate assessments of air quality.^8−10^ The detection of pollutants involves the nuanced measurement of gas concentrations, often in parts per million (ppm) or parts per billion (ppb), requiring sophisticated sensor technologies and signal processing methodologies. In healthcare applications, gas sensors play a crucial role in patient care by providing continuous and reliable monitoring of respiratory gases.^11−14^ They obey stringent accuracy standards, ensuring precise measurement of oxygen and carbon dioxide levels. Integrated into medical devices and patient monitoring systems, these sensors contribute to the maintenance of optimal gas concentrations in clinical environments.^15,16^ In essence, the technical sophistication of gas sensors is a testament to their multifaceted utility across various domains, where precision, reliability, and real-time responsiveness are paramount.

The detection of nitrogen dioxide (NO_2_) and ozone (O_3_) is important in various contexts due to their potential health and environmental impacts.^17^ Here are some specific reasons for the need for NO_2_ and O_3_ gas detectors.^13,18−20^ Both NO_2_ and O_3_ are air pollutants that can have adverse effects on human health. NO_2_ is a respiratory irritant, and long-term exposure can lead to respiratory problems. Ozone, while beneficial in the upper atmosphere, can be harmful when present at the ground level, causing respiratory issues and aggravating pre-existing conditions. Monitoring of the NO_2_ and the level of the O_3_ is often necessary to ensure compliance with environmental regulations. Industries and urban areas may have emission limits for these gases to minimize their impact on air quality. In certain industries, the production or use of chemicals can lead to the release of aqueous NO_2_ or O_3_. Gas detectors are essential for ensuring the safety of workers by providing early warnings in the event of gas leaks.^21^ NO_2_ is a common component of vehicle emissions, especially in urban areas, with heavy traffic. Monitoring NO_2_ levels helps assess the impact of traffic-related pollution and implement measures to reduce it.

In workplaces where there is a potential for NO_2_ or O_3_ emissions, gas detectors help maintain indoor air quality and protect the health of workers. Gas detectors for NO_2_ and O_3_ are crucial in emergency response situations such as chemical spills or leaks. Rapid detection allows for timely evacuation and containment measures. Researchers and environmental scientists use NO_2_ and O_3_ detectors to study air quality, pollution sources, and the impact of these gases on ecosystems and human health. Monitoring NO_2_ and O_3_ levels in populated areas helps in issuing public health alerts when concentrations reach levels that could cause health risks, especially for vulnerable populations. Ozone high in the earth’s atmosphere (stratosphere) protects life on earth by absorbing ultraviolet (UV) radiation. Monitoring ozone levels is important for understanding and protecting the ozone layer.^20,22,23^

In the present study, a ternary metal sulfide, combined with metal oxides, was synthesized by employing a hydrothermal synthesis method. The resultant nanocomposite material exhibits remarkable capabilities in the adsorption of two distinct gases at ambient temperature. Furthermore, this synthesized material demonstrates enhanced dual-detection properties for oxidizing gases under ambient conditions. Additionally, the surface and electronic characteristics of this nanocomposite have been rigorously analyzed and computed utilizing density functional theory (DFT) simulations, offering a comprehensive understanding of its adsorptive and sensory mechanisms at the molecular level.

## Experimental Section

### Materials and Methods

The heterostructure based on ternary metal sulfide and metal oxide was synthesized by using a one-step hydrothermal method. Lead chloride (PbCl_2_ purity >99%) was purchased from Sigma-Aldrich. Tin chloride (SnCl_4_·5H_2_O purity >99%) and thiourea were purchased from Alfa Aesar. The other chemical was also purchased from high grade and used without any further purification.

### Synthesis of PbSnS/SnO_2_

**Figure 1:**
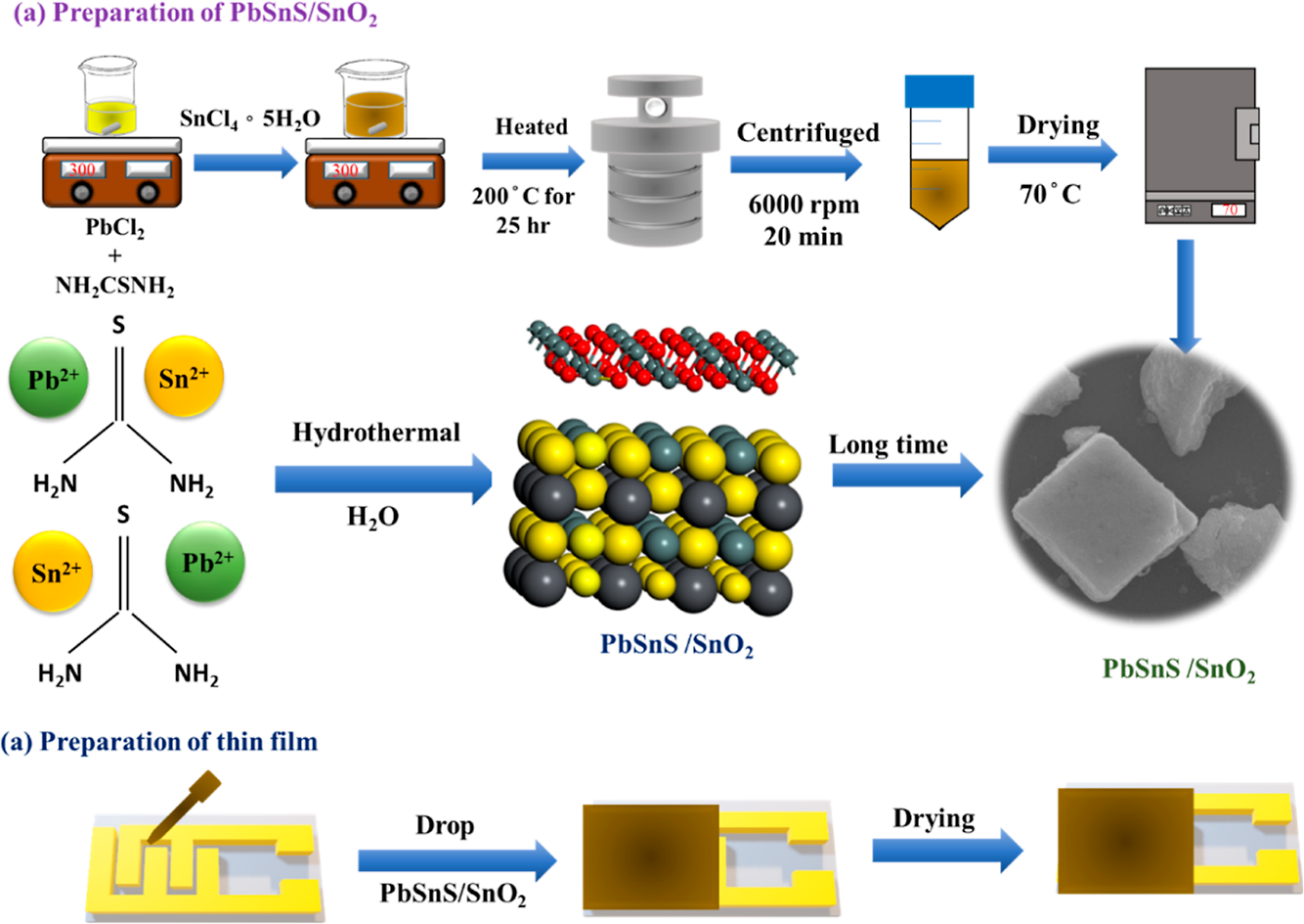
Schematic diagram for the synthesis of the PbSnS/SnO_2_ nanocomposite.

Initially, a one-step hydrothermal synthesis method was employed for the synthesis of ternary metal sulfide-incorporated metal oxide PbSnS/SnO_2_ composite materials. The synthesis involves the addition of lead chloride, tin chloride, and thiourea to 85 mL of deionized water, followed by magnetic stirring at room temperature for 20 to 30 min until complete dissolution of the solids. The resulting solution was transferred to a 100 mL high-pressure autoclave and subjected to a hydrothermal treatment at 200 °C for 25 h, followed by gradual cooling to room temperature. The resultant product was filtered by multiple centrifugation cycles using deionized water at 6000 rpm. The resulting precipitate was then dried at 70 °C to yield the PbSnS/SnO_2_ composite material as shown in Figure 1a. The chemical reaction involved in this process is given by eqs 1 and 2.
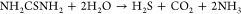
1
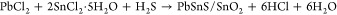
2

This one-step hydrothermal synthesis method offers distinct advantages, characterized by its cost-effectiveness, simplicity, and prevention of potential contamination that may arise during separate synthesis processes for PbSnS and SnO_2_. Notably, this method promotes a more homogeneous blending of the two semiconductor materials, facilitating the formation of heterojunction structures between their particles. The formation of such heterojunctions is advantageous for promoting efficient charge transfer at the interface and mitigating self-aggregation tendencies observed in the individual semiconductor particles. Subsequently, sensor devices are fabricated by incorporating the PbSnS/SnO_2_ heterostructure into a deionized water solvent and drop-casting the mixture onto a fork-shaped electrode as shown in Figure S1. Following drying on a heating plate at 80 °C, the PbSnS/SnO_2_ sensor is successfully used for further application.

### Characterization Details

Elemental mapping and SEM images were acquired by employing a field emission scanning electron microscope integrated with an energy-dispersive X-ray spectrophotometer (EDX). TEM images were captured by utilizing a Tecnai G2 microscope with a 200 kV accelerating voltage. X-ray diffraction (XRD) measurements were conducted by using a diffractometer (PANalytical B. V., The Netherlands) and CuKα radiation in the 10 to 80° degree range.

### Gas Sensing Setup

Gas sensing assessments were performed utilizing a custom gas sensing configuration, as reported in our previously published work,^24^ in which the pure gas was mixed with the atmospheric gas in the gas mixing chamber before being injected into the measurement system. The 2B Tech Model 714 has maintained the gas concentration of ppb order.^25^ When the film is exposed to various concentrations of NO_2_, a Keithley electrometer Model No. 2400 is used for the measurement of resistance with respect to time at 1 V. All the gas sensing measurements were taken at 300 K and relative humidity ∼65% RH.

### Computational Details

A theoretical model of PbSnS/SnO_2_ was created utilizing GaussView 06^26^ visualization software and subjected to computational analysis using the Gaussian 16^27^ software package. DFT^28^ calculations were employed, employing the LANL2DZ basis set to accurately describe the electronic structure and properties of the heterostructure. Subsequently, density of state (DOS) plots were generated using GaussSum software,^29^ providing visual representations of the electronic density distribution and energy states within the PbSnS/SnO_2_ heterostructure. This comprehensive computational approach facilitated the elucidation of electronic properties and behaviors, offering valuable insights into the material’s structural and electronic characteristics.

## Results and Discussion

**Figure 2:**
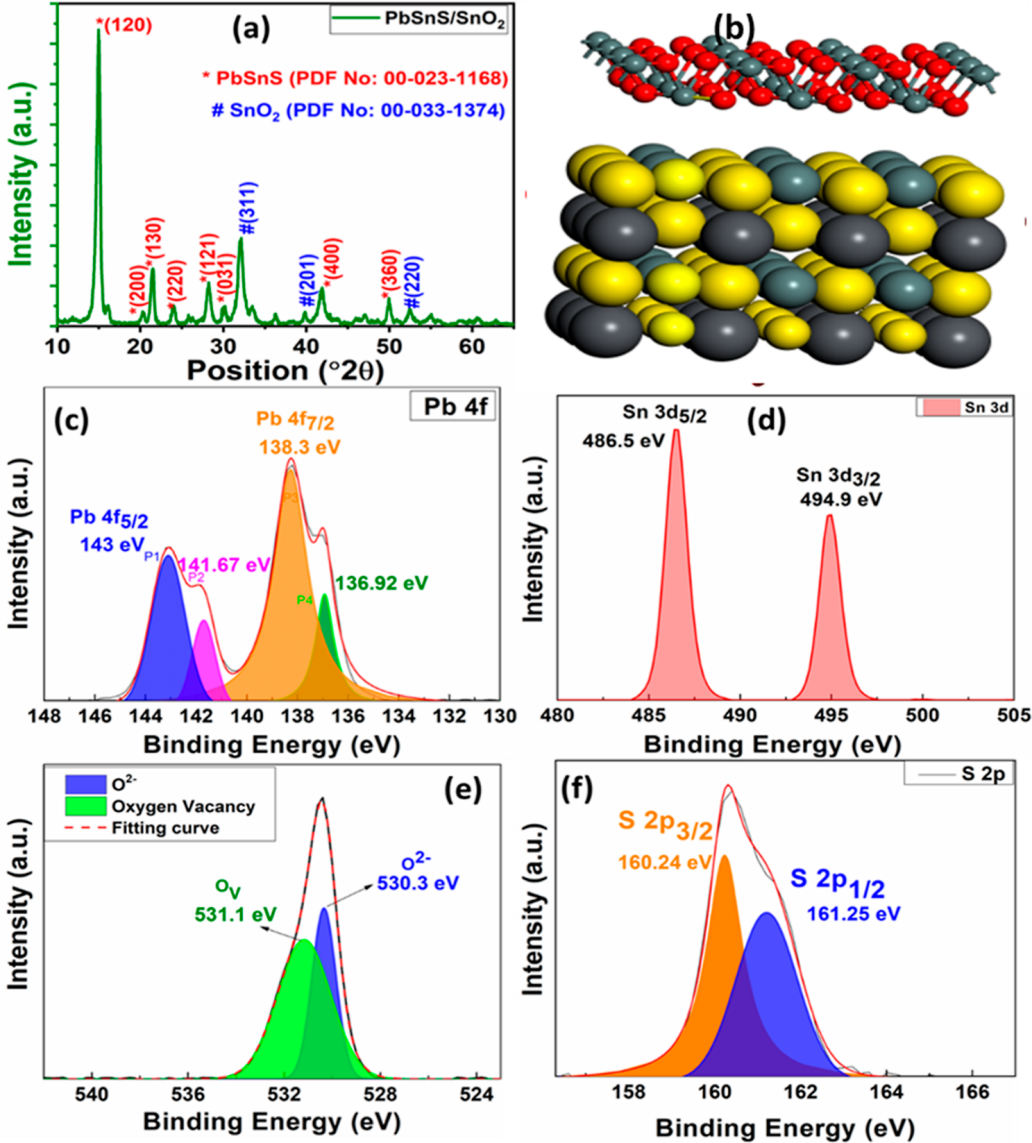
(a) XRD analysis of the PbSnS/SnO_2_ nanocomposite. (b) Crystal structure of PbSnS/SnO_2_. XPS peak fitting analysis of (c) Pb, (d) Sn, (e) O, and (f) S.

XRD analysis was performed on the PbSnS/SnO_2_ composite material as shown in Figure 2a. This analytical technique allows for the clarification of the crystalline structure and phase composition of the composite. Through XRD analysis, the characteristic diffraction patterns corresponding to the crystallographic phases present in the PbSnS/SnO_2_ composite were identified. This comprehensive characterization provides valuable insights into the structural properties and phase purity of the composite material, supporting the understanding of its potential applications and performance in gas sensing and other fields. The peaks at 16.1, 20.4, 21.45, 23.9, 28.6, 30.3, 41.3, and 49.9 belong to the (120), (200), (130), (220), (121), (031), (400), and (360) characteristics planes of PbSnS. Furthermore, the peaks at 31.5, 40.8, and 51.5 belong to the (111), (201), and (220) planes of SnO_2_. All the characteristic peaks perfectly match with the PbSnS JCPDS number 00–023–1168 and SnO_2_ JCPDS number 00–033–1374, respectively.

X-ray photoelectron spectroscopy (XPS) analysis was conducted on the PbSnS/SnO_2_ nanocomposite to investigate its surface chemistry and elemental composition shown in Figure 2c–f. The peak at 143 eV corresponds to the Pb 4f_7/2_ orbital. This peak typically represents metallic lead or lead in a lower oxidation state. The peak at 141.67 eV corresponds to the Pb 4f_5/2_ orbital. It may arise from lead oxides or other compounds containing lead in a higher oxidation state. The peak at 138.3 eV is associated with the Pb 4d_5/2_ orbital. This peak may arise from lead compounds or complexes with different coordination environments. The peak at 136.92 eV corresponds to the Pb 4d_3/2_ orbital. Similar to the previous peak, it may also arise from lead compounds or complexes with varying oxidation states and coordination environments. Overall, the presence of multiple peaks at different binding energies suggests the coexistence of lead in various chemical states or environments within the PbSnS material. Furthermore, the peak at 486.5 eV may correspond to the Sn 3d_5/2_ orbital. This peak typically represents metallic Sn or Sn in a lower oxidation state. The peak at 494.9 eV may correspond to the Sn 3d_3/2_ orbital. It may arise from tin oxides or other compounds containing tin in a higher oxidation state.

The peak at 531.1 eV for O corresponds to the 1s orbital. This peak typically represents oxygen atoms in a lower oxidation state or environments with weaker chemical bonding. The peak at 530.3 eV for O also corresponds to the O 1s orbital. However, it may arise from oxygen atoms in a higher oxidation state or environments with stronger chemical bonding, such as oxides or oxygen-containing functional groups. The peak at 160.24 eV for S corresponds to the S 2p_3/2_ orbital. This peak typically represents sulfur atoms in a lower oxidation state or environments with weaker chemical bonding. The peak at 161.25 eV for S also corresponds to the S 2p_3/2_ orbital. However, it may arise from sulfur atoms in a higher oxidation state or environments with stronger chemical bonding, such as sulfides or sulfur-containing functional groups.

**Figure 3:**
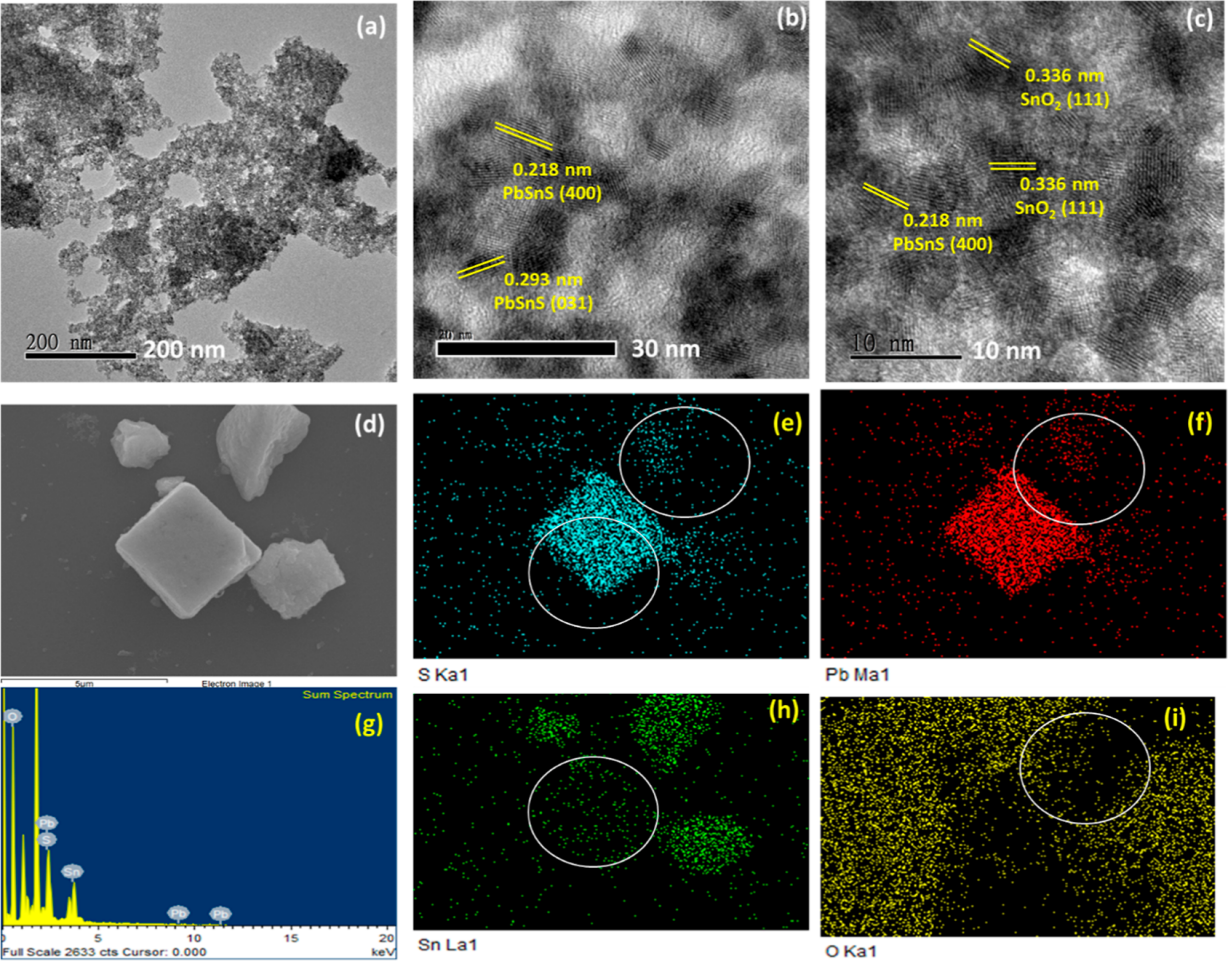
(a) High-resolution transmission electron microscopy (HRTEM) analysis of the PbSnS/SnO_2_ nanocomposite. (b,c) Lattice fringe analysis of the nanocomposite. (d–i) EDX analysis and elemental mapping of the PbSnS/SnO_2_ nanocomposite.

The surface morphology and lattice fringes have been analyzed by using SEM and TEM analysis and are depicted in Figure 3. The heterostructure based on PbSnS/SnO_2_ has been illustrated in Figure 3a in which the SnO_2_ nanoparticles are attached to the surface of PbSnS ternary metal sulfide. Furthermore, the lattice fringe having an interplanar spacing of 0.334 nm belongs to the (110) plane of SnO_2_ and 0.341 and 0.345 nm belong to the (111) and (112) plane of PbSnS, respectively, as shown in Figure 3b,c. Moreover, the elemental analysis of PbSnS/SnO_2_ has been depicted in Figure 3d–i. On comparing Figure 3d,e,g it has been observed that SnO_2_ makes heterojunctions with PbSnS. Furthermore, high EDX peaks having high intensity for Pb, Sn, S, and O confirm the formation of the PbSnS/SnO_2_ nanocomposite.

**Figure 4:**
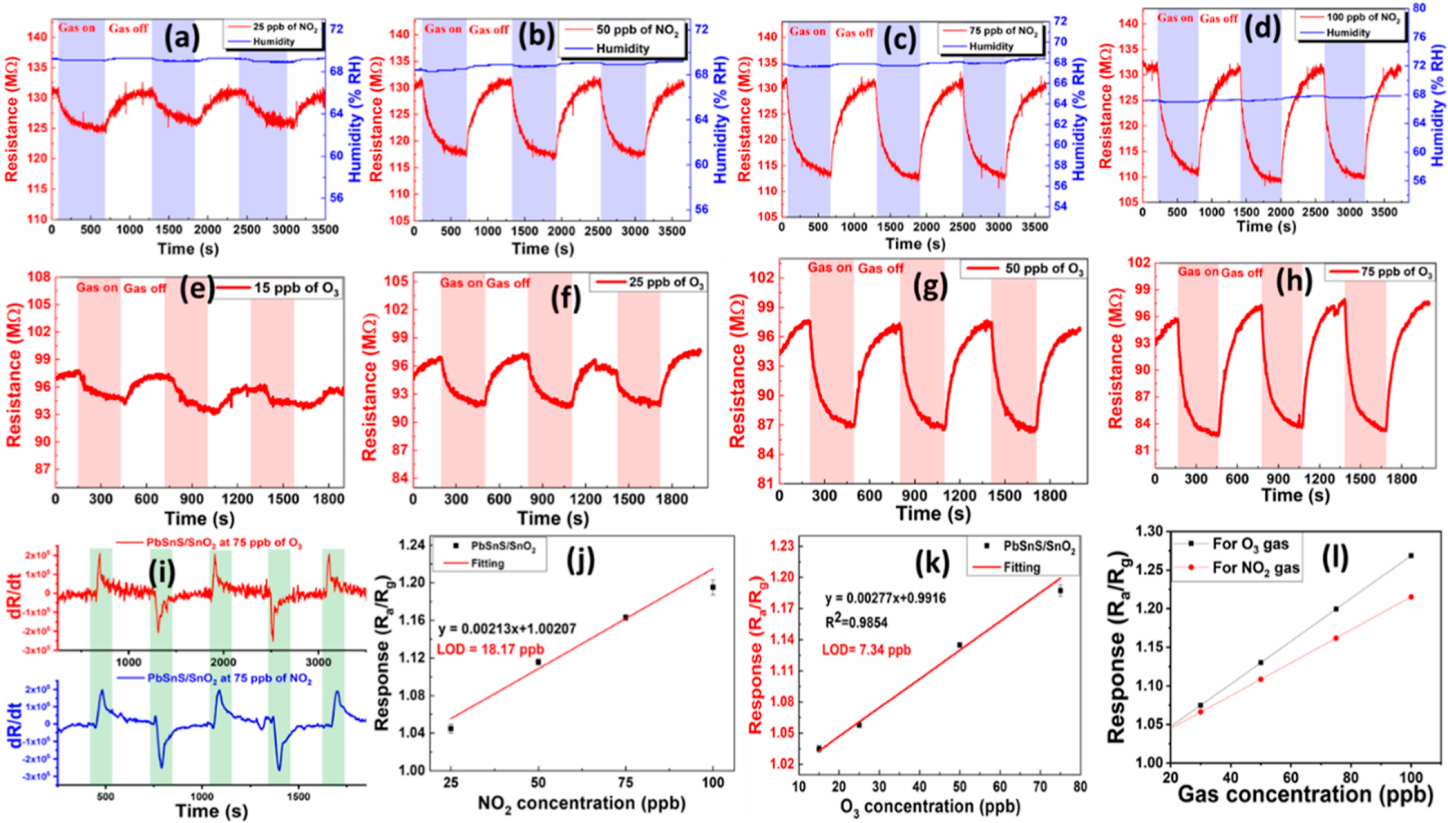
Variation in the resistance of the PbSnS/SnO_2_ thin film in the presence of NO_2_ (a) at 25 ppb, (b) at 50 ppb, (c) at 75 ppb, and (d) at 100 ppb, in the presence of O_3_ (e) at 15 ppb, (f) at 25 ppb, (g) at 50 ppb, and (h) at 75 ppb. (i) Differential change of sensor response at 75 ppb of NO_2_ and O_3_. (j,k) Linear fitted graph of NO_2_ and O_3_. (l) Comparison in the slope of sensor response for both gases.

The gas sensing performance of the PbSnS/SnO_2_ nanocomposite was thoroughly characterized. Dynamic variations in resistance were observed upon exposure to varying concentrations of NO_2_ and O_3_ gases, as illustrated in Figure 4a–h. Upon the formation of the heterostructure, interactions with atmospheric oxygen led to the generation of oxygen species on the thin film surface. Consequently, upon interaction with oxidizing gases such as NO_2_ and O_3_, charge transfer occurs from NO_2_ to the PbSnS/SnO_2_ composite. Given the p-type properties of the heterostructure, a decrease in the resistance was observed. Notably, during NO_2_ gas testing, resistance variations were found to increase with increasing gas concentrations. Specifically, the gas sensing response exhibited an escalation from 1.04 to 1.20 as gas concentrations increased from 25 to 100 ppb.

The transient dynamic resistance curve indicates that the PbSnS/SnO_2_-based gas sensor effectively adsorbs oxidizing gases, such as NO_2_ and O_3_, resulting in a measurable change in the resistance of the thin film. To enhance the sensor’s selectivity toward these high oxidizing gases, the adsorption and desorption rates were calculated and are illustrated in Figure 4i. The results demonstrate that the adsorption rate of the adsorption of O_3_ is significantly higher than that of the adsorption rate of NO_2_. Consequently, leveraging the differential response at identical concentrations of NO_2_ and O_3_ enables the real-time detection of both gases with an improved accuracy.

The gas sensing characteristics for ozone (O_3_) sensing are illustrated in Figure 4e–h. These curves depict a higher variation in resistance in the presence of O_3_ compared to NO_2_ gas. The sensor response increases from 1.03 to 1.23 with changing concentrations from 15 to 75 ppb. These findings validate the dual gas detection capability of the PbSnS/SnO_2_ nanocomposite. Additionally, the differential change of gas sensing response for NO_2_ and O_3_ has been depicted in Figure 4i. The limit of detection (LOD) was calculated using linear fitting curves for NO_2_ and O_3,_ as depicted in Figure 4j,k. The LOD for NO_2_ and O_3_ gas was determined to be 16.17 and 7.84 ppb, respectively.

**Figure 5:**
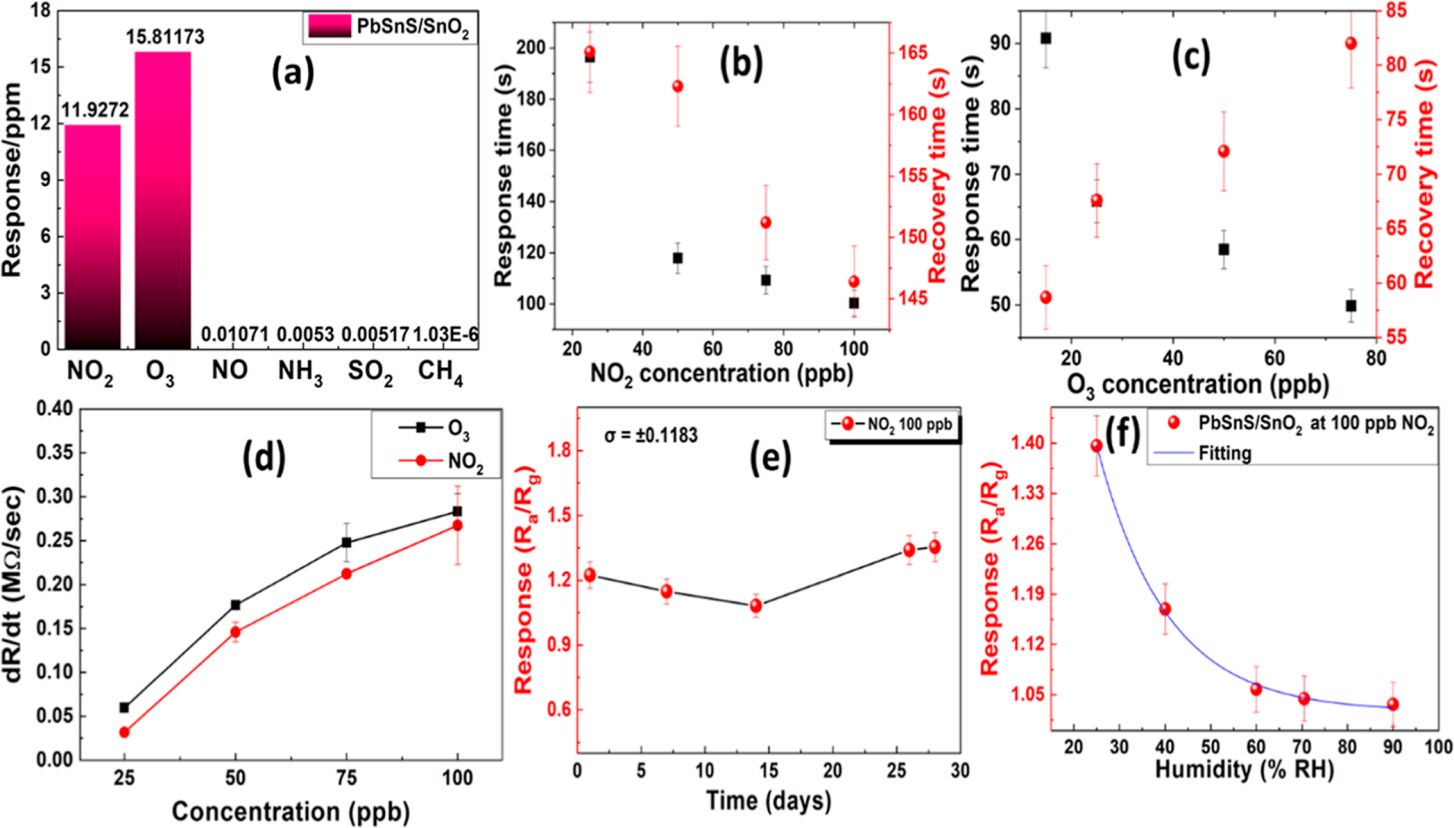
(a) Selectivity test of the PbSnS/SnO_2_ nanocomposite, (b,c) response and recovery time at different concentrations of NO_2_ and O_3_, (d) comparison of sensor response of NO_2_ and O_3_, (e) long-term stability test in the presence of NO_2_, and (f) effect of humidity on sensor response.

The sensor selectivity test is depicted in Figure 5a. The result reveals that the PbSnS/SnO_2_ nanocomposite has a high sensor response toward NO_2_ and O_3_ in comparison to other gases. The dynamic resistance curve of the PbSnS/SnO_2_ sensor under NO, CH_4_, SO_2_, and NH_3_ gases are depicted in Figures S2–S5. The response and recovery time of PbSnS/SnO_2_ for NO_2_ gas at different concentrations has been illustrated in Figure 5b. From these figures, it has been found that response and recovery time decrease on increasing concentrations because the higher concentration increases the surface charge potential and large charge transfer rate. Therefore, the adsorption and desorption rates increase with increasing concentrations. Furthermore, if we consider the response and recovery time for O_3_ gas, we found that the response time decreases and the recovery time increases because due to having high oxidation energy of O_3_, molecules are strongly attached. So, during the desorption process, the O_3_ molecules are attached to the defected sites of PbSnS/SnO_2_. Therefore, the desorption process takes time, and it increases by increasing the gas concentrations.

Furthermore, the comparison of response for NO_2_ and O_3_ gas at different concentrations has been depicted in Figure 5d. After analyzing the gas sensing response, it has been observed that the response increases linearly but O_3_ has more response at lower concentrations in comparison to NO_2_. Moreover, the long-term stability was also tested by using NO_2_ gas, and the sensor response is approximately similar after 30 days as shown in Figure 5e. In real-time gas sensing applications, humidity also plays a very important role in gas adsorption. Therefore, we also analyze the gas sensing performance at different humidity levels, depicted in Figure 5f. The result reveals that the PbSnS/SnO_2_ sensor response decreases with increasing humidity because at higher humidity, the water molecule covers the thin film so there is less interaction between the gas molecule and the thin film. The NO_2_ response curve of the PbSnS/SnO_2_ sensor under different humidity levels has been illustrated in Figure S6.

The comparative analysis of PbSnS/SnO_2_-based NO_2_ and O_3_ sensors with previously reported sensors is presented in Table 1, revealing the superior dual gas sensing capabilities of the PbSnS/SnO_2_ sensor. Notably, the PbSnS/SnO_2_ sensor exhibits heightened performance metrics in comparison with other sensors. Furthermore, a notable advantage of the PbSnS/SnO_2_ sensor lies in its operational feasibility at room temperature, making it suitable for real-time gas detection applications.

**Table 1:** Comparative Study of the PbSnS/SnO_2_ Sensor with Previously Published Work

S. no	materials	type of gas	concentration	operating temperature	sensor response	response/recovery time	ref
1	IGZO-ZnO	NO_2_	5 ppm	250	48	172/295	(30)
2	rGO-SnS_2_	NO_2_	5 ppm	150	32	50/48	(31)
3	MoS_2_/PbS	NO_2_	10 ppm	RT	6.15	15/62	(32)
4	SnO_2_/MoS_2_	NO_2_	10 ppm	RT	0.28	400/180	(8)
5	SnS_2_@SnO_2_	NO_2_	0.2 ppm	RT	5.3	950/1160	(33)
6	CuAlO_2_	O_3_	200 ppb	200	1.9	29/45	(34)
7	β-In_2_S_3_	O_3_	40 ppb	160	1.5	147/414	(35)
8	ZnO–SnO_2_	O_3_	20 ppb	RT/UV	8		(36)
9	a-ZTO	O_3_	5 ppm	RT	12.8	62/91	(37)
10	PbSnS/SnO_2_	NO_2_	25 ppb	RT	1.04	197/165	this work
11	PbSnS/SnO_2_	O_3_	15 ppb	RT	1.03	91/29	this work

Furthermore, the gas sensing mechanism of the PbSnS/SnO_2_ heterostructure involves the phenomenon of adsorption and charge transfer of oxygen species. Upon exposure to ambient air, oxygen molecules adhere to the junctions of the heterostructure, generating oxygen species. Subsequently, when the heterostructure encounters oxidizing gases, such as NO_2_ and O_3_, electron exchange occurs between the heterostructure and the oxygen species. Specifically, the heterostructure accepts electrons from the O^2–^ molecules, resulting in the release of free electrons within the PbSnS/SnO_2_ material. This electron transfer process leads to an increase in the conductivity of the heterostructure, facilitating the detection of oxidizing gases.

**Figure 6:**
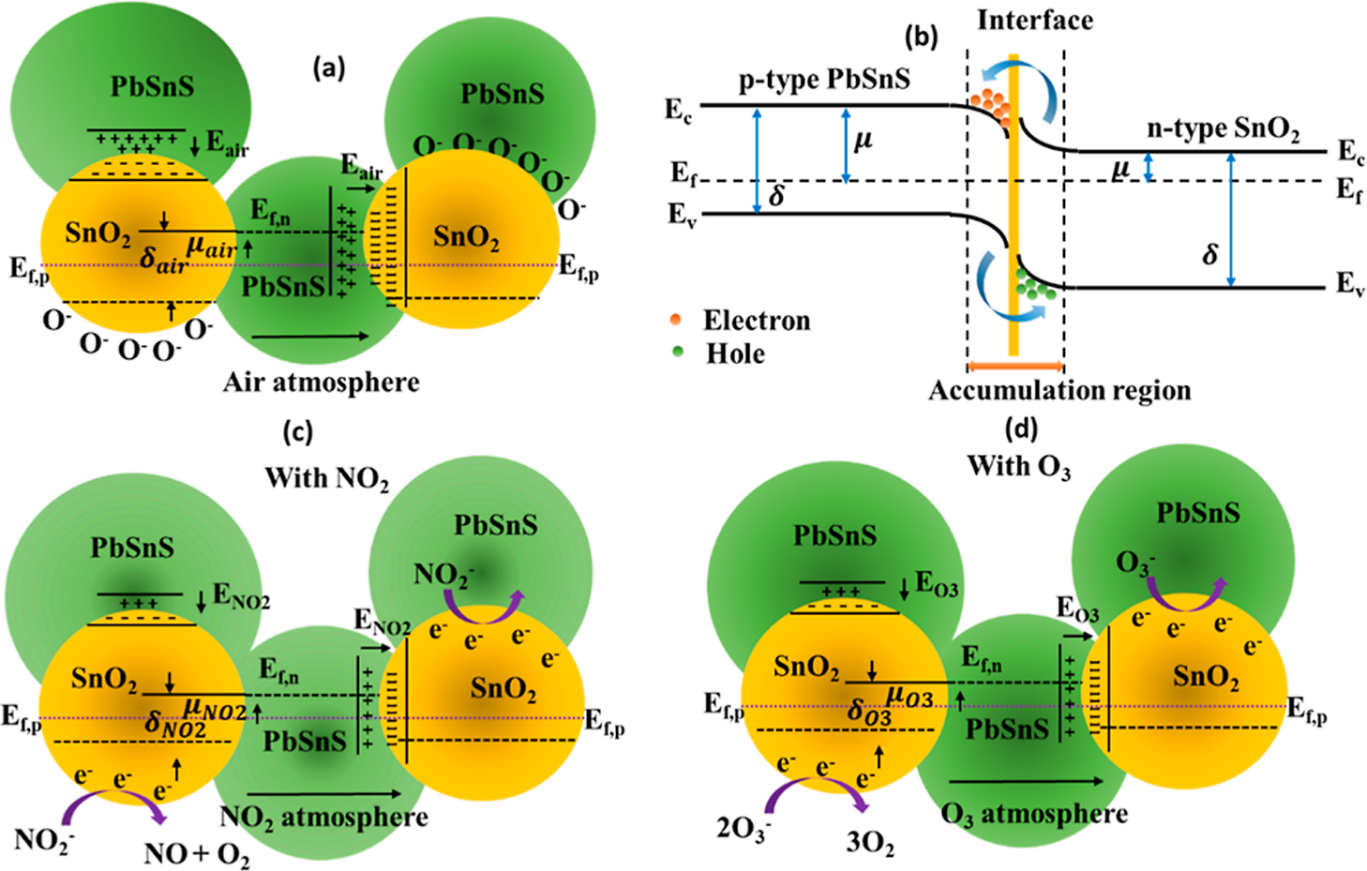
(a) Formation of oxygen species along the junction and on the nanocomposite surface. (b) Band diagram of PbSnS/SnO_2_. Change in the energy level after interaction with (c) NO_2_ and (d) O_3_.

The gas sensing mechanism of PbSnS/SnO_2_ in response to NO_2_ and O_3_ gases is shown in Figure 6a–d. This graphical representation unveils shifts in band levels that are consequential to interactions with these oxidizing gases. Upon exposure to oxygen molecules, a localized potential gradient emerges across the junction, as depicted in Figure 6a. This phenomenon is aptly elucidated through the band diagram presented in Figure 6b, where discernible accumulation regions appear along the heterojunction grain boundaries. These potential fluctuations across the junctions are instrumental in modulating the conductivity characteristics of the materials.

Figure 6c illustrates the perturbations in energy levels within PbSnS/SnO_2_ upon interaction with NO_2_. This dynamic process involves NO_2_ molecules assimilating electrons from the surrounding atmosphere, subsequently transferring them to PbSnS/SnO_2_ post-interaction with oxygen species. This electron exchange results in the conversion of NO_2_ into NO, as shown by eqs 3–5.
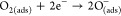
3
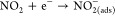
4
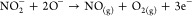
5

Similarly, during the interaction with ozone (O_3_) molecules, the highly oxidizing ozone species undergo ionization by acquiring electrons from the surrounding atmosphere. Subsequently, these ionized ozone molecules adsorb onto the oxygen species present on the surface of PbSnS/SnO_2_, as illustrated in Figure 6d. Furthermore, upon interaction with oxygen species, the ionized ozone molecules donate electrons to the PbSnS/SnO_2_ material, facilitating their conversion into oxygen molecules (O_2_). This electron exchange process is represented by eqs 6 and 7. Equation 6 depicts the ionization of ozone molecules through the acquisition of electrons, while eq 7 represents the subsequent electron transfer from the ionized ozone molecules to the PbSnS/SnO_2_ surface, leading to the formation of oxygen molecules.
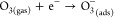
6
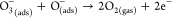
7

Furthermore, a theoretical model has been developed to analyze the charge transfer and gas adsorption mechanism of PbSnS/SnO_2_. In this section, we calculate the adsorption energy at different sites to analyze which atom interaction is responsible for such adsorption. The different theoretical parameters have been calculated by using the value of higher occupied molecular orbitals (HOMO) and lower unoccupied molecular orbitals (LUMO) as reported in our previously published papers.^24,38,39^ The variation in different theoretical parameters after interaction with NO_2_ on different atoms has been depicted in Table 2.

**Table 2:** Variation in Different Theoretical Parameters of PbSnS/SnO_2_ before and after Interaction with NO_2_ on Various Sites

theoretical parameters	before interaction	after interaction with NO_2_					
		SnO_2_	Pb_(junction)_	S_(junction)_	Pb	Sn	S
adsorption energy (eV)		–0.08	–0.79	–0.26	–0.05	–0.27	–0.24
ionization potential (eV)	4.925	4.906	4.954	4.818	4.953	4.664	4.69
electron affinity (eV)	3.201	3.256	3.872	3.168	3.868	2.765	2.73
HOMO–LUMO gap (eV)	1.724	1.65	1.082	1.65	1.105	1.89	1.96
sensor response		0.043	0.37	0.043	0.35	–0.09	–0.13
electronegativity (eV)	4.063	4.081	4.061	3.993	4.410	3.714	3.71
dipole moment (debye)	3.11	3.20	3.18	4.81	3.92	3.42	4.30

From the data in Table 2, it is clear that the lead (Pb) atom located at the junction is the most important in the adsorption of the NO_2_ gas molecules. When compared with other atoms, the Pb atom shows an adsorption energy of −0.76 eV and a sensor response of 0.37, indicating its key role in the process. Experimental investigations further corroborate these findings, indicating a reduction in the width of the accumulation layer during NO_2_ adsorption. This reduction consequently leads to an enhancement in the conductivity of the material. Moreover, theoretical data give the variation of the HOMO–LUMO gap after NO_2_ adsorption on the Pb atom. Specifically, a decrease in the HOMO–LUMO gap is observed after interaction with the NO_2_ molecules. This reduction in the gap signifies diminished hindrance for electron mobility within the material, thereby contributing to the observed increase in the conductivity of PbSnS/SnO_2_.

**Figure 7:**
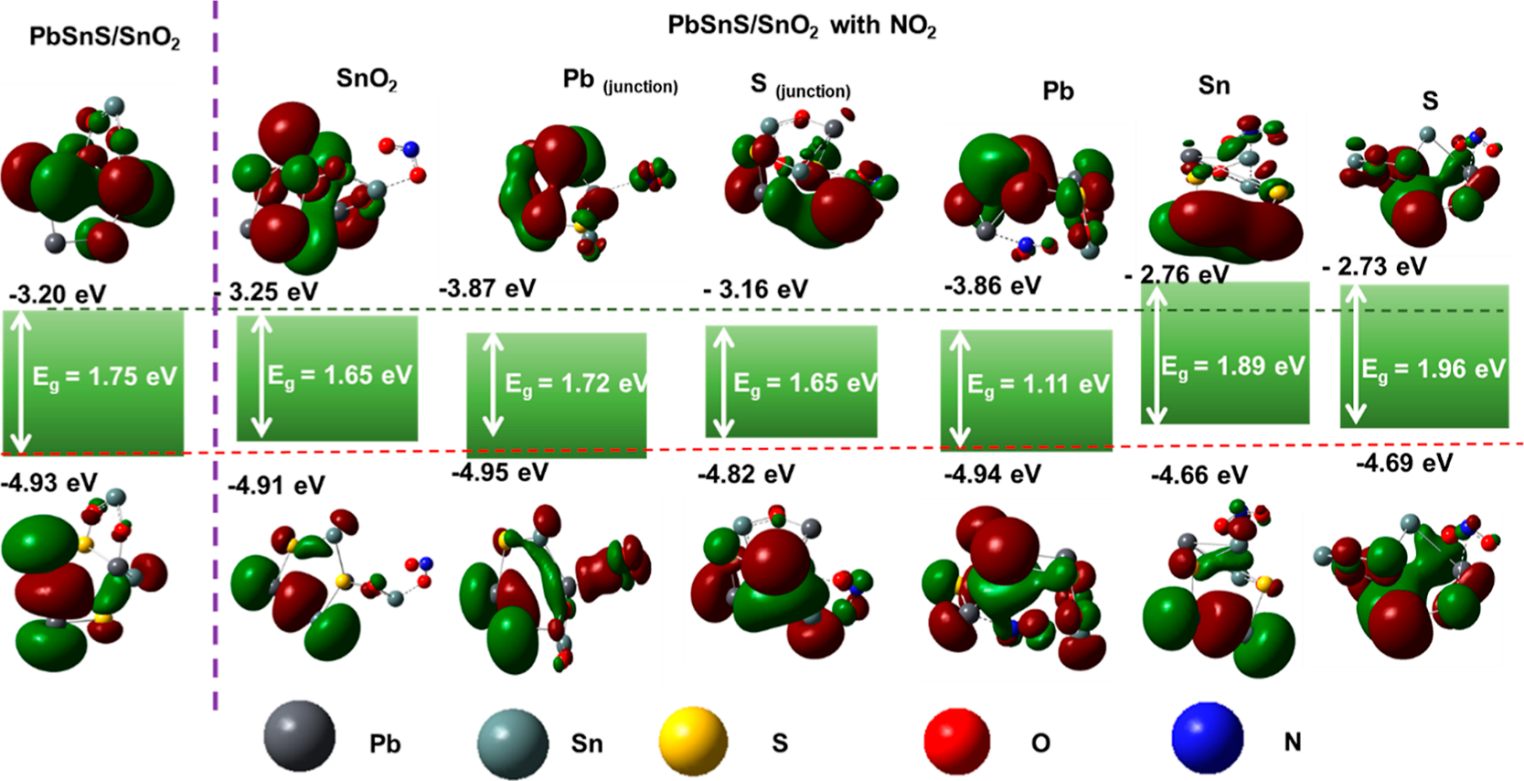
Variation in the band level of PbSnS/SnO_2_ before and after adsorbing NO_2_ at different sites.

The variation in the HOMO–LUMO levels of PbSnS/SnO_2_ before and after interaction with NO_2_ has been illustrated in Figure 7. In this figure, the positive charge density has been illustrated by green color and negative by red color, respectively. Specifically, the plot exhibits a noticeable decrease in the HOMO–LUMO gap compared to its preinteraction state. This reduction signifies a diminishing energy barrier for the electron movement within the material. Consequently, electrons encounter less resistance, leading to an increase in the material’s conductivity. Such changes in the HOMO–LUMO plot reflect the dynamic electronic restructuring induced by NO_2_ interaction, elucidating the mechanism underlying the observed conductivity enhancement in PbSnS/SnO_2_ heterostructures.

Furthermore, the interaction of the O_3_ molecules causes significant changes in the energy levels of the PbSnS/SnO_2_ heterostructures. Analyzing the theoretical parameters after O_3_ adsorption, as shown in Table 3, revealed notable differences at various sites within the material. Specifically, SnO_2_ demonstrates increased activity in the adsorption of the O_3_ molecules, indicating that this part of the heterostructure plays a dominant role in the O_3_ sensing process. Quantitatively, the adsorption energy of O_3_ on SnO_2_ is determined to be −0.22 eV, accompanied by a sensor response of 0.479. This indicates a strong affinity of SnO_2_ sites for O_3_ adsorption, underscoring their significant role in mediating the interaction between the material and ozone molecules. These findings highlight the preferential reactivity and adsorption capability of SnO_2_ sites toward O_3_, emphasizing their importance in facilitating O_3_ sensing applications and elucidating the mechanistic pathways underlying the material’s response to ozone exposure.

**Table 3:** Variation in Different Theoretical Parameters of PbSnS/SnO_2_ before and after Interaction with O_3_ on Various Sites

theoretical parameters	before	after interaction with O_3_					
		SnO_2_	Pb_(junction)_	S_(junction)_	Pb	Sn	S
adsorption energy (eV)		–0.22	–0.20	–0.09	–0.29	–0.10	–0.33
ionization potential (eV)	4.925	4.692	4.362	4.224	4.75	4.74	4.668
electron affinity (eV)	3.201	3.794	3.086	3.728	3.20	3.18	2.811
HOMO–LUMO gap (eV)	1.724	0.898	1.276	0.496	1.55	1.56	1.875
sensor response		0.479	0.259	0.712	0.10	0.095	–0.087
electronegativity (eV)	4.063	4.243	3.724	3.976	3.975	3.96	3.73
dipole moment (debye)	3.11	1.813	3.118	3.776	4.149	2.685	3.934

**Figure 8:**
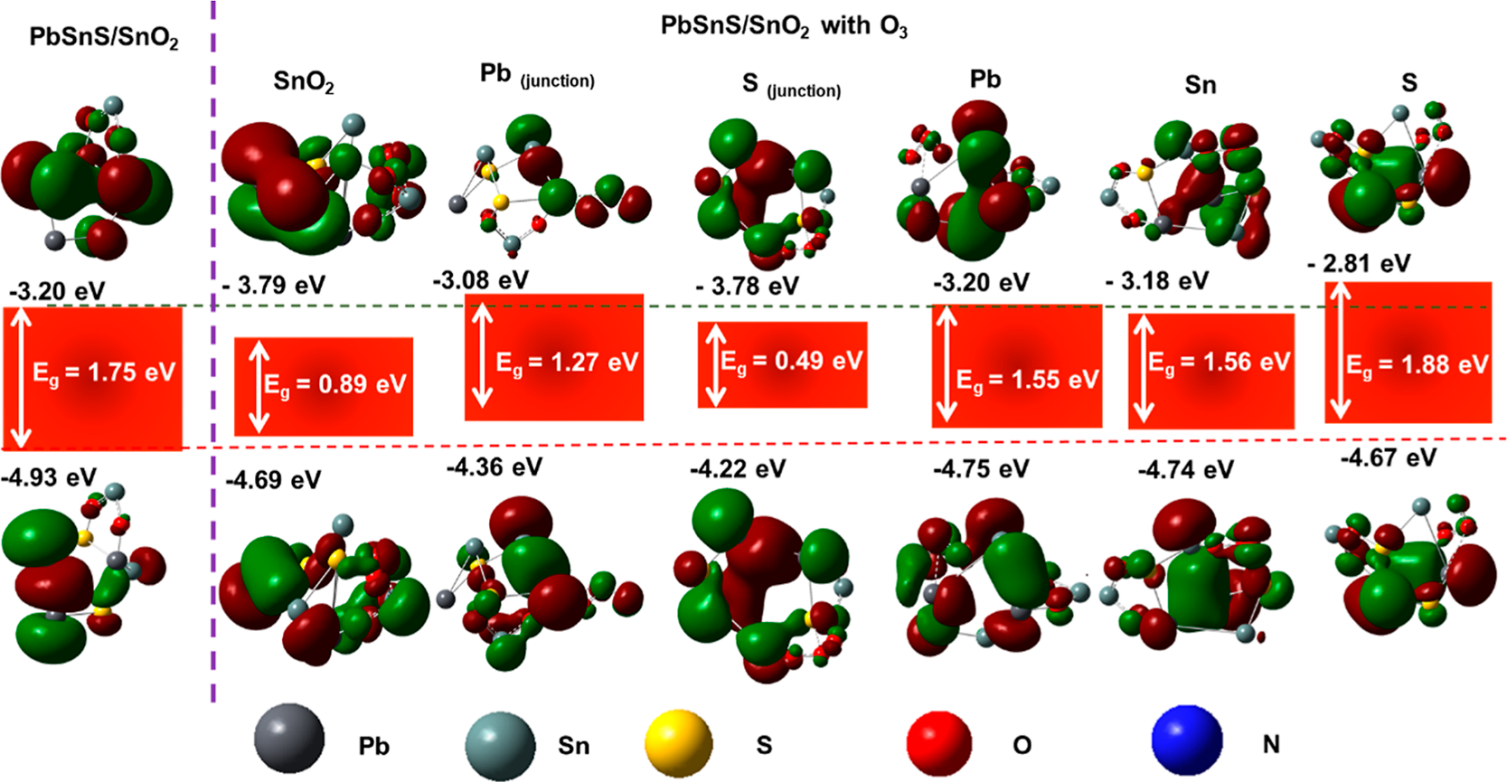
Variation in the band level of PbSnS/SnO_2_ before and after adsorbing O_3_ at different sites.

The interaction of O_3_ at different sites on the PbSnS/SnO_2_ heterostructure induces variations in the band levels, elucidating the impact of O_3_ adsorption on the material’s electronic structure and also shown in Figure 8. In this figure, the positive charge density is depicted using green coloration, while the negative charge density is represented by red color. Before O_3_ adsorption, the band levels of PbSnS/SnO_2_ are characteristic of its pristine state, exhibiting distinct energy levels corresponding to different sites within the heterostructure. Upon O_3_ adsorption, alterations in the band levels occur, reflecting changes in the electronic configuration and energy states of the material.

These variations may include shifts in the valence band maximum (VBM) and conduction band minimum (CBM) at specific sites where the O_3_ molecules interact with the heterostructure. For instance, at sites where SnO_2_ is highly active for O_3_ adsorption, the band levels may experience pronounced shifts compared with other sites. This indicates a significant modification in the electronic structure induced by the O_3_ interaction, potentially leading to changes in the material’s conductivity and sensing properties. Detailed characterization of these band level variations provides crucial insights into the electronic response of PbSnS/SnO_2_ to O_3_ exposure, facilitating the understanding of its ozone sensing mechanism and informing the design of efficient ozone detection devices.

**Figure 9:**
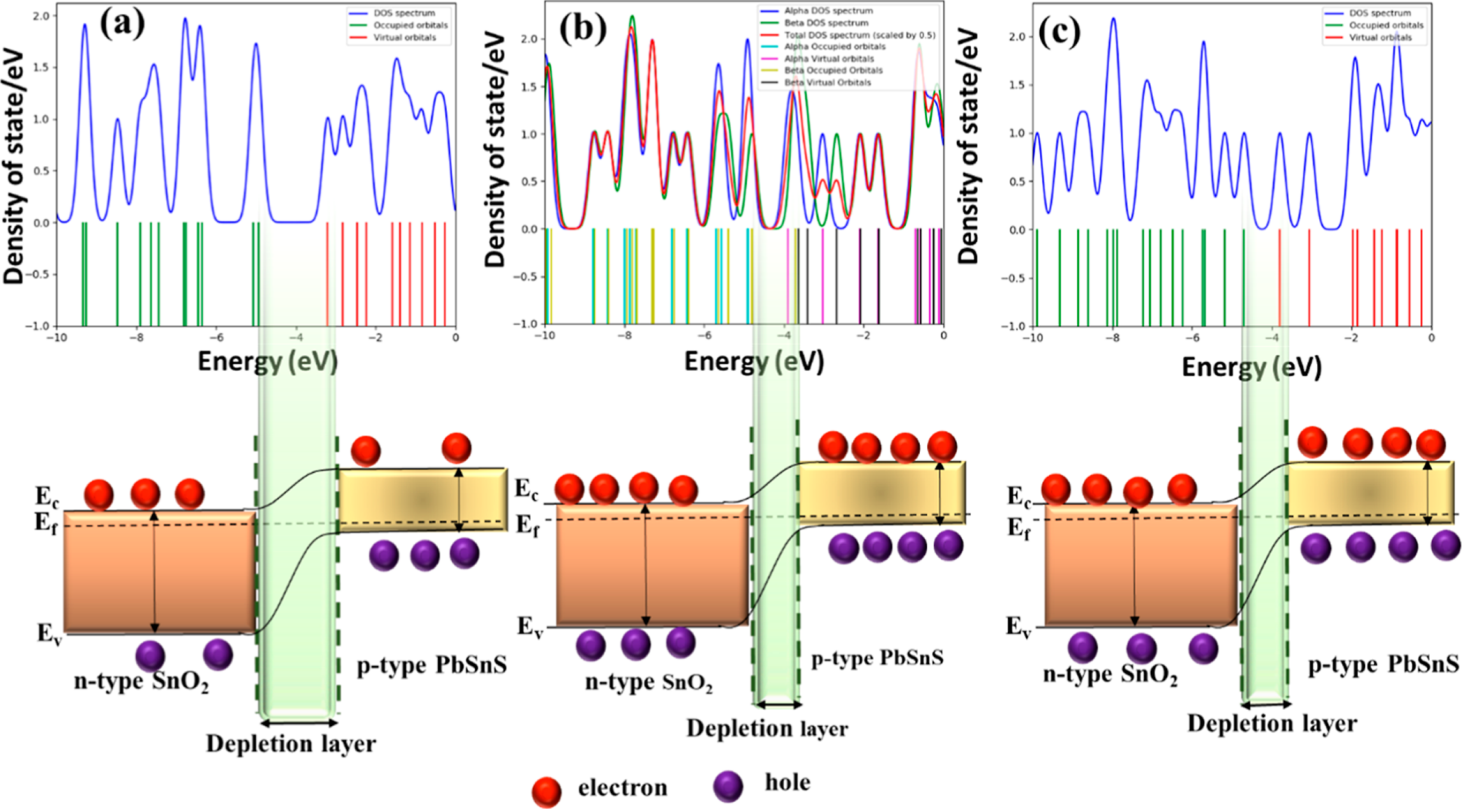
(a) DOS analysis of PbSnS/SnO_2_ before and after interaction of (b) NO_2_ and (c) O_3_.

The DOS analysis of PbSnS/SnO_2_ before and after interaction with NO_2_ and O_3_ reveals significant changes in the electronic structure of the material as shown in Figure 9a–c. Before interaction with these gases, the DOS exhibits characteristic peaks and features corresponding to the electronic states of PbSnS/SnO_2_ in its pristine state. Upon interaction with NO_2_ and O_3_, alterations in the DOS profiles are observed, indicating modifications in the density and distribution of electronic states within the material. These changes may include shifts in peak positions, broadening or narrowing of energy bands, and the appearance or disappearance of specific features in the DOS spectra.

In the case of the NO_2_ interaction, the DOS analysis may reveal changes associated with the adsorption of NO_2_ molecules on specific sites within the PbSnS/SnO_2_ heterostructure, leading to variations in the electronic structure near the adsorption sites. Similarly, interaction with O_3_ can induce alterations in the DOS profiles, reflecting the impact of adsorption of O_3_ on the electronic states and band structure of PbSnS/SnO_2_. These changes may arise from charge transfer phenomena, modifications in band alignment, or structural rearrangements induced by the O_3_ interaction. Overall, DOS analysis provides valuable insights into the electronic properties and behavior of PbSnS/SnO_2_ before and after exposure to NO_2_ and O_3_, facilitating the understanding of their gas sensing mechanisms and the optimization of sensing device performance.

## Conclusions

In summary, the successful synthesis of the PbSnS/SnO_2_ heterostructure via a one-step hydrothermal method is confirmed by various characterization techniques. XRD analysis demonstrates the precise matching of characteristic peaks with those of PbSnS and SnO_2_, validating the formation of the heterostructure. HRTEM imaging and elemental mapping further corroborate the formation of the heterostructure. Moreover, the fabricated thin film based on PbSnS/SnO_2_ exhibits excellent real-time sensing capabilities toward NO_2_ and O_3_ gases. The sensor response for NO_2_ gas reaches 1.04 at 25 ppb, with a LOD of 18.17 ppb. Similarly, for the O_3_ gas, the sensor response is 1.03 at 15 ppb, with an LOD of 7.34 ppb. Additionally, long-term stability testing and humidity sensing experiments demonstrate the sensor’s robustness over extended durations and its reliable performance across a wide range of humidity levels. Furthermore, DFT calculations corroborated the sensing mechanism, elucidating that the Pb atom in PbSnS/SnO_2_ is primarily responsible for the adsorption of NO_2_ gas, whereas SnO_2_ in PbSnS/SnO_2_ is responsible for the adsorption of O_3_ gas. These findings not only validate the efficacy of the PbSnS/SnO_2_ heterostructure for gas sensing applications but also contribute to a deeper understanding of the fundamental principles governing gas adsorption processes.

## Supporting Information Available

Detailed schematic diagram of the interdigital electrode configuration used in the sensing device; gas sensing response curves for various target gases, illustrating the device’s performance; and analysis of the variation in gas sensing response under different humidity conditions, offering insights into the sensor’s reliability and environmental adaptability (PDF)

## References

[ref1] Kuretake T.; Kawahara S.; Motooka M.; Uno S. An Electrochemical Gas Biosensor Based on Enzymes Immobilized on Chromatography Paper for Ethanol Vapor Detection. Sensors 2017, 17 (2), 281 10.3390/s17020281. 28157154 PMC5336011

[ref2] Peera S. G.; Koutavarapu R.; Chao L.; Singh L.; Murugadoss G.; Rajeshkhanna G. 2D MXene Nanomaterials as Electrocatalysts for Hydrogen Evolution Reaction (HER): A Review. Micromachines 2022, 13 (9), 1499 10.3390/mi13091499. 36144122 PMC9500977

[ref3] Zhu S.; Khan M. A.; Kameda T.; Xu H.; Wang F.; Xia M.; Yoshioka T. New Insights into the Capture Performance and Mechanism of Hazardous Metals Cr3+ and Cd2+ onto an Effective Layered Double Hydroxide Based Material. J. Hazard. Mater. 2022, 426, 128062 10.1016/j.jhazmat.2021.128062. 34929593

[ref4] Onyancha R. B.; Ukhurebor K. E.; Aigbe U. O.; Osibote O. A.; Kusuma H. S.; Darmokoesoemo H.; Balogun V. A. A Systematic Review on the Detection and Monitoring of Toxic Gases Using Carbon Nanotube-Based Biosensors. Sens. Bio-Sensing Res. 2021, 34, 100463 10.1016/j.sbsr.2021.100463.

[ref5] Kumar U.; Yadav B. C.; Huang W.-M.; Wu C.-H. Metal oxide-based nanocomposites designed for humidity sensor applications. In Complex and Composite Metal Oxides for Gas VOC and Humidity Sensors; Elsevier, 2024; Vol. 1, pp 331–346.

[ref6] Rodner M.; Eriksson J. First-Order Time-Derivative Readout of Epitaxial Graphene-Based Gas Sensors for Fast Analyte Determination. Sens. Actuator. Rep. 2020, 2 (1), 100012 10.1016/j.snr.2020.100012.

[ref7] Sonker R. K.; Yadav B. C.; Gupta V.; Tomar M. Fabrication and Characterization of ZnO-TiO2-PANI (ZTP) Micro/Nanoballs for the Detection of Flammable and Toxic Gases. J. Hazard. Mater. 2019, 370, 126–137. 10.1016/j.jhazmat.2018.10.016. 30528466

[ref8] Cui S.; Wen Z.; Huang X.; Chang J.; Chen J. Stabilizing MoS 2 Nanosheets through SnO 2 Nanocrystal Decoration for High-Performance Gas Sensing in Air. Small 2015, 11 (19), 2305–2313. 10.1002/smll.201402923. 25641557

[ref9] Ma M.; Yang X.; Ying X.; Shi C.; Jia Z.; Jia B. Applications of Gas Sensing in Food Quality Detection: A Review. Foods 2023, 12 (21), 3966 10.3390/foods12213966. 37959084 PMC10648483

[ref10] Feng D.; Du L.; Xing X.; Wang C.; Chen J.; Zhu Z.; Tian Y.; Yang D. Highly Sensitive and Selective NiO/WO 3 Composite Nanoparticles in Detecting H 2 S Biomarker of Halitosis. ACS Sens. 2021, 6 (3), 733–741. 10.1021/acssensors.0c01280. 33528988

[ref11] Kumar R.; Goel N.; Hojamberdiev M.; Kumar M. Transition Metal Dichalcogenides-Based Flexible Gas Sensors. Sens. Actuators, A 2020, 303, 111875 10.1016/j.sna.2020.111875.

[ref12] Kumar U.; Hsieh H.-W.; Liu Y.-C.; Deng Z.-Y.; Chen K.-L.; Huang W.-M.; Wu C.-H. Revealing a Highly Sensitive Sub-Ppb-Level NO 2 Gas-Sensing Capability of Novel Architecture 2D/0D MoS 2/SnS Heterostructures with DFT Interpretation. ACS Appl. Mater. Interfaces 2022, 14 (28), 32279–32288. 10.1021/acsami.2c03173. 35818995

[ref13] Cheng L.-H.; Kumar U.; Deng Z.-Y.; Wu C.-H. Improved Charge Transfer for NO 2 Gas Sensors by Using 0D SnS Quantum Dot/2D WSe 2 Heterostructures. ACS Appl. Nano Mater. 2023, 6 (11), 9506–9514. 10.1021/acsanm.3c01181.

[ref14] Kumar U.; Wu C.-H.; Singh K.; Yadav B. C.; Huang W.-M. Low-Dimensional Advanced Functional Materials as Hazardous Gas Sensing. In Advanced Functional Materials for Optical and Hazardous Sensing: Synthesis and Applications; Springer, 2023; pp 31–45. 10.1007/978-981-99-6014-9_2.

[ref15] He W.; Qian Y.; Lee B. S.; Zhang F.; Rasheed A.; Jung J.-E.; Kang D. J. Ultrahigh Output Piezoelectric and Triboelectric Hybrid Nanogenerators Based on ZnO Nanoflakes/Polydimethylsiloxane Composite Films. ACS Appl. Mater. Interfaces 2018, 10 (51), 44415–44420. 10.1021/acsami.8b15410. 30507129

[ref16] Zhang H.; Zhang D.; Mao R.; Zhou L.; Yang C.; Wu Y.; Liu Y.; Ji Y. MoS2-Based Charge Trapping Layer Enabled Triboelectric Nanogenerator with Assistance of CNN-GRU Model for Intelligent Perception. Nano Energy 2024, 127, 109753 10.1016/j.nanoen.2024.109753.

[ref17] Deng Z.-Y.; Lin W.-Y.; Kumar U.; Chen K.-L.; Wang T.-H.; Chen J.-H.; Wu C.-H. Atomic-Level Insights of Polypyrrole Grafted InGaZnO Structure for Ppb-Level Ozone Gas Sensing at Low Operating Temperature. ACS Appl. Mater. Interfaces 2024, 16 (31), 41495–41503. 10.1021/acsami.4c07392. 39069916

[ref18] Kumar U.; Hsieh H.-W.; Liu Y.-C.; Deng Z.-Y.; Chen K.-L.; Huang W.-M.; Wu C.-H. Revealing a Highly Sensitive Sub-Ppb-Level NO 2 Gas-Sensing Capability of Novel Architecture 2D/0D MoS 2/SnS Heterostructures with DFT Interpretation. ACS Appl. Mater. Interfaces 2022, 14, 32279 10.1021/acsami.2c03173. 35818995

[ref19] Kumar U.; Liu Y.-C.; Hsieh H.-W.; Deng Z.-Y.; Huang W.-M.; Wu C.-H. Revealing Ppb Order NO2 Adsorption Capability of 2D/0D p-p Heterojunctions Based on Sb/SnS Nanocomposite. J. Alloys Compd. 2023, 969, 172451 10.1016/j.jallcom.2023.172451.

[ref20] Kumar U.; Huang S.-M.; Deng Z. Y.; Yang C.-X.; Haung W. M.; Wu C.-H. Comparative DFT Dual Gas Adsorption Model of ZnO and Ag/ZnO with Experimental Applications as Gas Detection at Ppb Level. Nanotechnology 2021, 33, 105502 10.1088/1361-6528/ac3e2f. 34844230

[ref21] Kumar U.; Yadav B. C.; Kumar K.; Haldar T.; Kanth Kumar V. V. R.; Huang W.-M.; Wu C.-H. Exploring the Room Temperature Chemiresistive LPG and Humidity Sensing Properties of MWCNT/TiO2 Nanocomposite with DFT Interpretations. Sens. Actuators, A 2024, 379, 115851 10.1016/j.sna.2024.115851.

[ref22] daSilva L. F.; Catto A. C.; Avansi W.; Cavalcante L. S.; Andrés J.; Aguir K.; Mastelaro V. R.; Longo E. A Novel Ozone Gas Sensor Based on One-Dimensional (1D) α-Ag 2 WO 4 Nanostructures. Nanoscale 2014, 6 (8), 4058–4062. 10.1039/C3NR05837A. 24609437

[ref23] Lo T.-H.; Shih P.-Y.; Wu C.-H. The Response of UV/Blue Light and Ozone Sensing Using Ag-TiO2 Planar Nanocomposite Thin Film. Sensors 2019, 19 (23), 5061 10.3390/s19235061. 31756975 PMC6929171

[ref24] Kumar U.; Tsou Y.-C.; Deng Z.-Y.; Yadav B. C.; Huang W.-M.; Wu C.-H. Exploring the Weak Visible-near-Infrared and NO 2 Detection Capabilities of PbS/Sb 2 O 5 Heterostructures with DFT Interpretations. Nanotechnology 2024, 35 (26), 265501 10.1088/1361-6528/ad375d. 38527363

[ref25] Birks J. W.; Turnipseed A. A.; Andersen P. C.; Williford C. J.; Strunk S.; Carpenter B.; Ennis C. A. Portable Calibrator for NO Based on the Photolysis of N& Lt;Sub& Gt;2& Lt;/Sub& Gt;O and a Combined NO& Lt;Sub& Gt;2& Lt;/Sub& Gt;/NO/O& Lt;Sub& Gt;3& Lt;/Sub& Gt; Source for Field Calibrations of Air Pollution Monitors. Atmos. Meas. Technol. 2020, 13 (2), 1001–1018. 10.5194/amt-13-1001-2020.

[ref26] Dennington R.; Keith T. A.; Millam J. M. Gauss View 6; Semichem Inc., 2016.

[ref27] Frisch M. J.; Trucks G. W.; Schlegel H. B.; Scuseria G. E.; Robb M. A.; Cheeseman J. R.; Scalmani G.; Barone V.; Mennucci B.; Petersson G. A.; Nakatsuji H.; Caricato M.; Li X.; Hratchian H. P.; Izmaylov A. F.; Bloino J.; Zheng G.; Sonnenberg J. L.; Hada M.; Ehara M.; Toyota K.; Fukuda R.; Hasegawa J.; Ishida M.; Nakajima T.; Honda Y.; Kitao O.; Nakai H.; Vreven T.; Montgomery J. A. Jr.; Peralta J. E.; Ogliaro F.; Bearpark M.; Heyd J. J.; Brothers E.; Kudin K. N.; Staroverov V. N.; Kobayashi R.; Normand J.; Raghavachari K.; Rendell A.; Burant J. C.; Iyengar S. S.; Tomasi J.; Cossi M.; Rega N.; Millam J. M.; Klene M.; Knox J. E.; Cross J. B.; Bakken V.; Adamo C.; Jaramillo J.; Gomperts R.; Stratmann R. E.; Yazyev O.; Austin A. J.; Cammi R.; Pomelli C.; Ochterski J. W.; Martin R. L.; Morokuma K.; Zakrzewski V. G.; Voth G. A.; Salvador P.; Dannenberg J. J.; Dapprich S.; Daniels A. D.; Farkas Ö.; Foresman J. B.; Ortiz J. V.; Cioslowski J.; Fox D. J. Gaussian 16, Revision C.01; Gaussian, Inc.: Wallingford CT, 2019.

[ref28] AlRabiah H.; Muthu S.; Al-Omary F.; Al-Tamimi A.-M.; Raja M.; Muhamed R. R.; El-Emam A. A.-R. Molecular Structure, Vibrational Spectra, NBO, Fukui Function, HOMO-LUMO Analysis and Molecular Docking Study of 6-[(2-Methylphenyl)Sulfanyl]-5-Propylpyrimidine-2,4(1H,3H)-Dione. J. Chem. Chem. Eng. 2017, 36 (1), 59 10.20450/mjcce.2017.1001.

[ref29] O’boyle N. M.; Tenderholt A. L.; Langner K. M. Cclib: A Library for Package-Independent Computational Chemistry Algorithms. J. Comput. Chem. 2008, 29 (5), 839–845. 10.1002/jcc.20823. 17849392

[ref30] Eadi S. B.; Yan H.; Kumar P. S.; Rathinam Y.; Lee H.-D. IGZO-Decorated ZnO Thin Films and Their Application for Gas Sensing. Environ. Res. 2022, 214, 113796 10.1016/j.envres.2022.113796. 35810811

[ref31] Cheng M.; Wu Z.; Liu G.; Zhao L.; Gao Y.; Zhang B.; Liu F.; Yan X.; Liang X.; Sun P.; Lu G. Highly Sensitive Sensors Based on Quasi-2D RGO/SnS2 Hybrid for Rapid Detection of NO2 Gas. Sensor. Actuator. B Chem. 2019, 291, 216–225. 10.1016/j.snb.2019.04.074.

[ref32] Liu J.; Hu Z.; Zhang Y.; Li H.-Y.; Gao N.; Tian Z.; Zhou L.; Zhang B.; Tang J.; Zhang J.; Yi F.; Liu H. MoS2 Nanosheets Sensitized with Quantum Dots for Room-Temperature Gas Sensors. Nano-Micro Lett. 2020, 12 (1), 59 10.1007/s40820-020-0394-6. PMC7770826 34138314

[ref33] Liu D.; Tang Z.; Zhang Z. Visible Light Assisted Room-Temperature NO2 Gas Sensor Based on Hollow SnO2@SnS2 Nanostructures. Sensor. Actuator. B Chem. 2020, 324, 128754 10.1016/j.snb.2020.128754.

[ref34] Thirumalairajan S.; Mastelaro V. R. A Novel Organic Pollutants Gas Sensing Material P-Type CuAlO 2 Microsphere Constituted of Nanoparticles for Environmental Remediation. Sensor. Actuator. B Chem. 2016, 223, 138–148. 10.1016/j.snb.2015.09.092.

[ref35] Souissi R.; Bouguila N.; Bendahan M.; Aguir K.; Fiorido T.; Abderrabba M.; Halidou I.; Labidi A. Ozone Sensing Study of Sprayed β-In2S3 Thin Films. J. Alloys Compd. 2022, 900, 163513 10.1016/j.jallcom.2021.163513.

[ref36] daSilva L. F.; M’Peko J.-C.; Catto A. C.; Bernardini S.; Mastelaro V. R.; Aguir K.; Ribeiro C.; Longo E. UV-Enhanced Ozone Gas Sensing Response of ZnO-SnO2 Heterojunctions at Room Temperature. Sensor. Actuator. B Chem. 2017, 240, 573–579. 10.1016/j.snb.2016.08.158.

[ref37] Huang C.-Y.; Yan C.-Y.; Lou Y.-Q. Dual-Functional Hybrid ZnSnO/Graphene Nanocomposites with Applications in High-Performance UV Photodetectors and Ozone Gas Sensors. Ceram. Int. 2022, 48 (3), 3527–3535. 10.1016/j.ceramint.2021.10.131.

[ref38] Kumar U.; Yadav B. C. Development of Humidity Sensor Using Modified Curved MWCNT Based Thin Film with DFT Calculations. Sensor. Actuator. B Chem. 2019, 288, 399–407. 10.1016/j.snb.2019.03.016.

[ref39] Kumar U.; Yang Y.-H.; Deng Z.-Y.; Lee M.-W.; Huang W.-M.; Wu C.-H. In Situ Growth of Ternary Metal Sulfide Based Quantum Dots to Detect Dual Gas at Extremely Low Levels with Theoretical Investigations. Sensor. Actuator. B Chem. 2022, 353, 131192 10.1016/j.snb.2021.131192.

