# Supplementary material for: Interfacial Engineering Facilitates Real-Time Detection of Dual Hazardous Gases at ppb Levels via Single-Step Hydrothermal Nanoarchitectonics of Self-Assembled PbSnS/SnO2 Heterostructures
Source: ACS Sens. 2025 Jan 30;10(3):2019–29. doi: 10.1021/acssensors.4c03215 (PMC11959608; doi:10.1021/acssensors.4c03215)
Supplement: Supplementary file 1 — se4c03215_si_001.pdf [file se4c03215_si_001.pdf]

Interfacial Engineering Facilitates Real-Time Detection of Dual Hazardous Gases at ppb Levels via Single-Step Hydrothermal Nanoarchitectonics of Self-Assembled PbSnS/SnO_2 Heterostructures

Utkarsh Kumar[a], Yu-Wen Yeh[b], Zu-Yin Deng[a], Wen-Min Huang[a*] and Chiu-Hsien Wu[a,b*]

[a]Department of Physics, National Chung Hsing University, Taichung 402, Taiwan

[b]Institute of Nanoscience, National Chung Hsing University, Taichung 402, Taiwan

*correspondence: Chiu-Hsien Wu (chwu@phys.nchu.edu.tw) and Wen-Min Huang(wenmin@phys.nchu.edu.tw)

Table of content

Fig. S1 Schematic diagram of interdigital electrode used for making sensor device

Fig. S2 Dynamic resistance curve of PbSnS/SnO_2 thin film in the presence of 100 ppm of NO.

Fig. S3 Dynamic resistance curve of PbSnS/SnO_2 thin film in the presence of CH_4

Fig. S4 Dynamic resistance curve of PbSnS/SnO_2 thin film in the presence of 200 ppm of SO_2

Fig. S5 Dynamic resistance curve of PbSnS/SnO_2 thin film in the presence of 100 ppm of NH_3

Fig. S6 (a-f) NO_2 sensing response at different levels of humidity

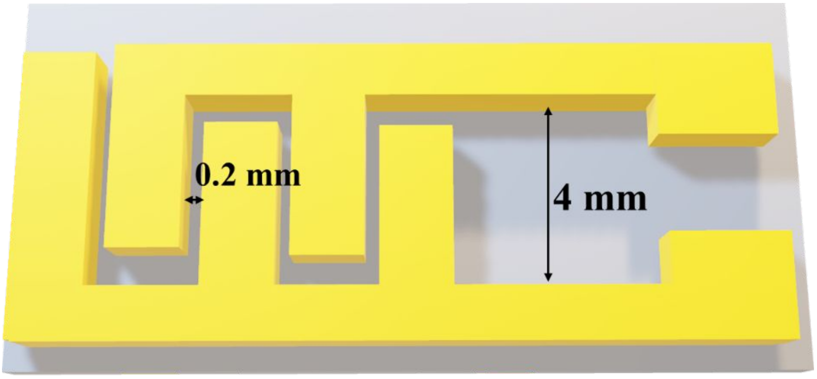

Figure S1 Schematic diagram of interdigital electrode used for making sensor device

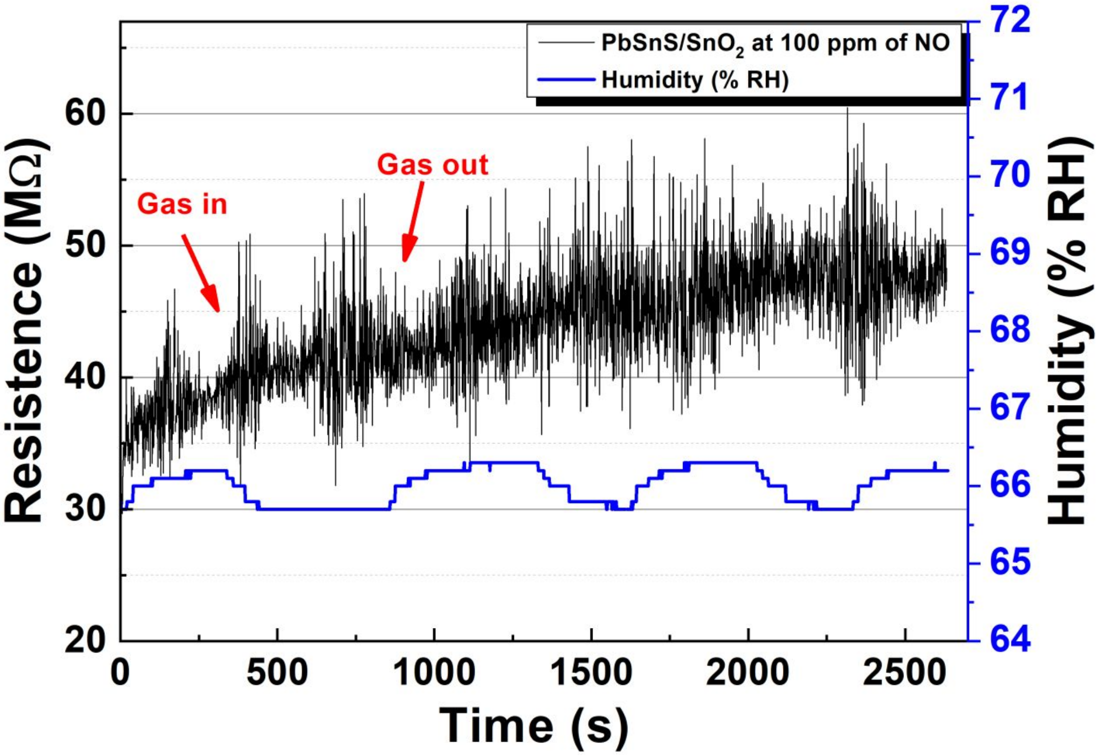

Fig. S2 Dynamic resistance curve of $PbSnS/SnO_2$ thin film in the presence of 100 ppm of NO

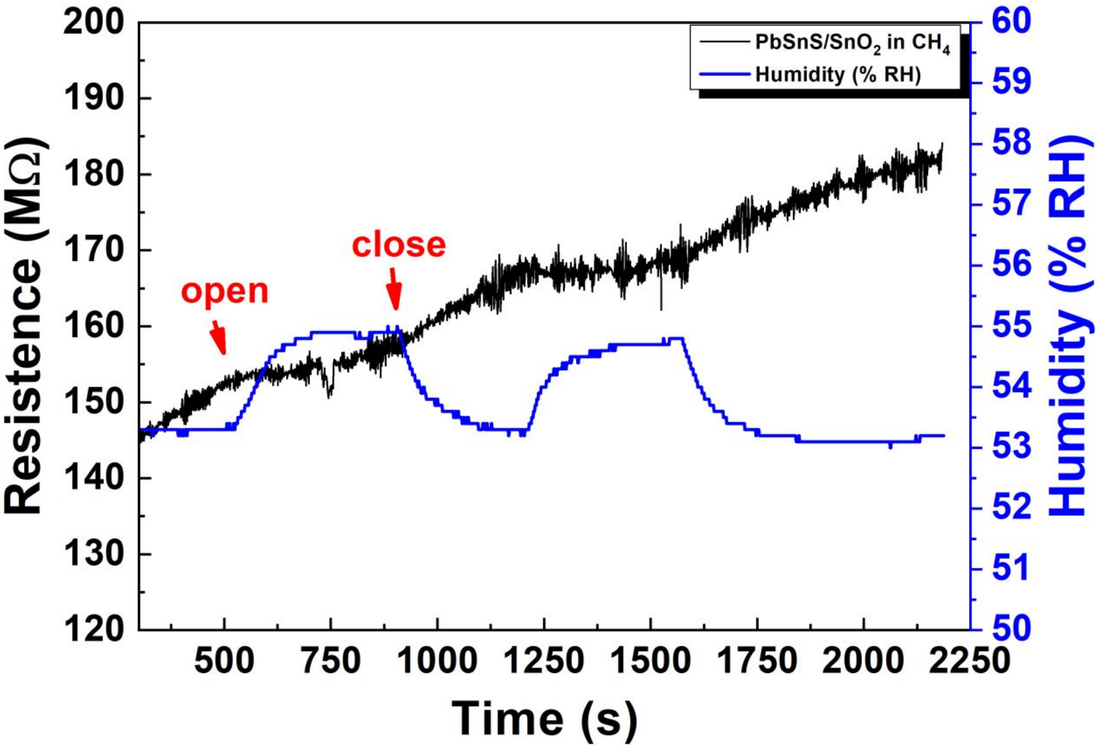

Fig. S3 Dynamic resistance curve of $PbSnS/SnO_2$ thin film in the presence of CH_4

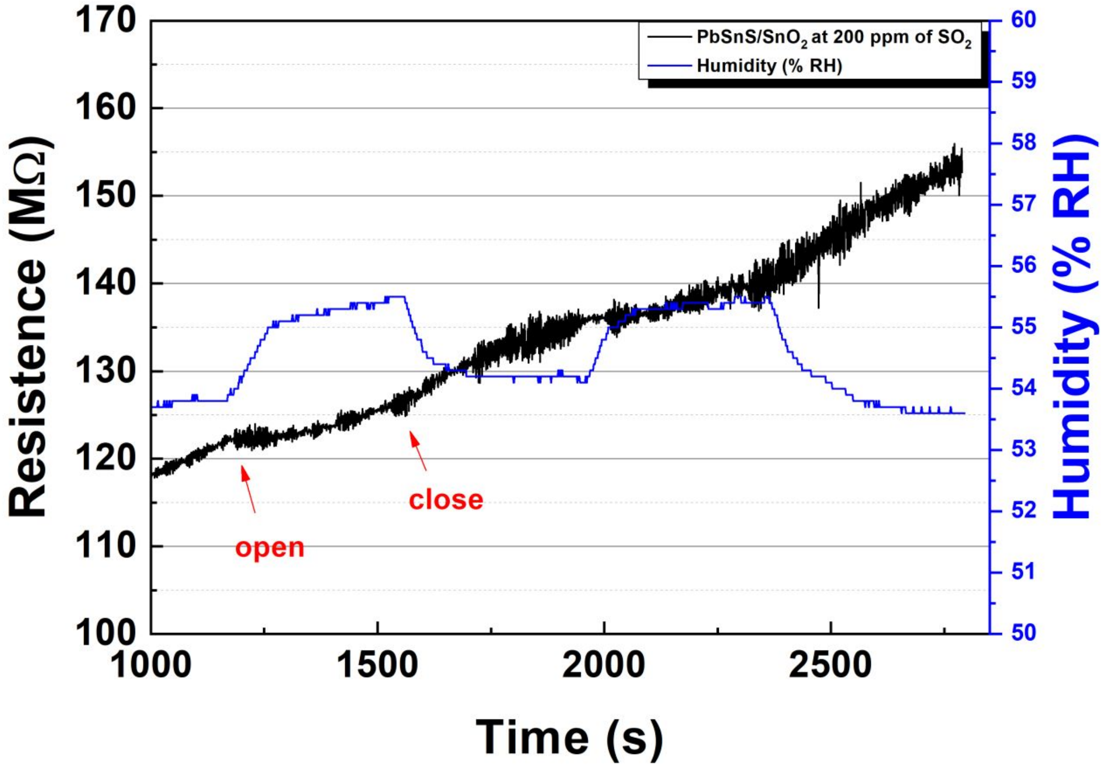

Fig. S4 Dynamic resistance curve of PbSnS/SnO_2 thin film in the presence of 200 ppm of SO_2

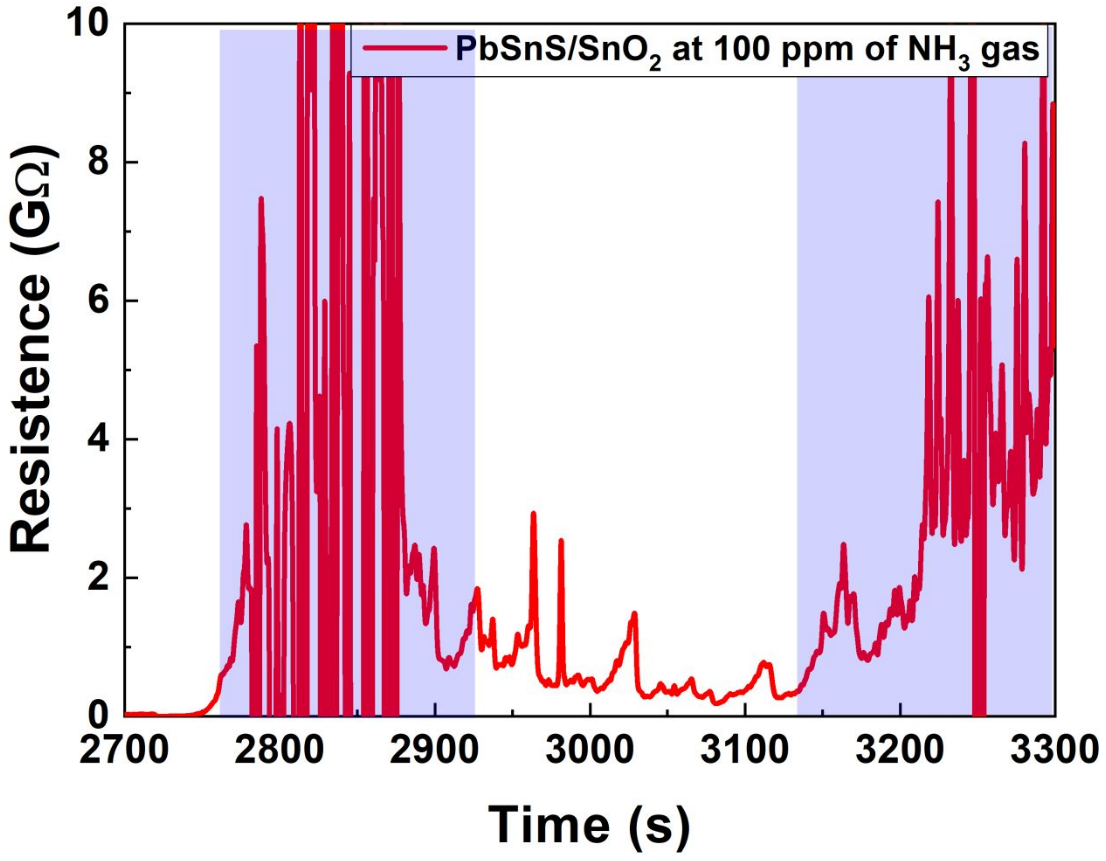

Fig. S5 Dynamic resistance curve of PbSnS/SnO_2 thin film in the presence of 100 ppm of NH_3

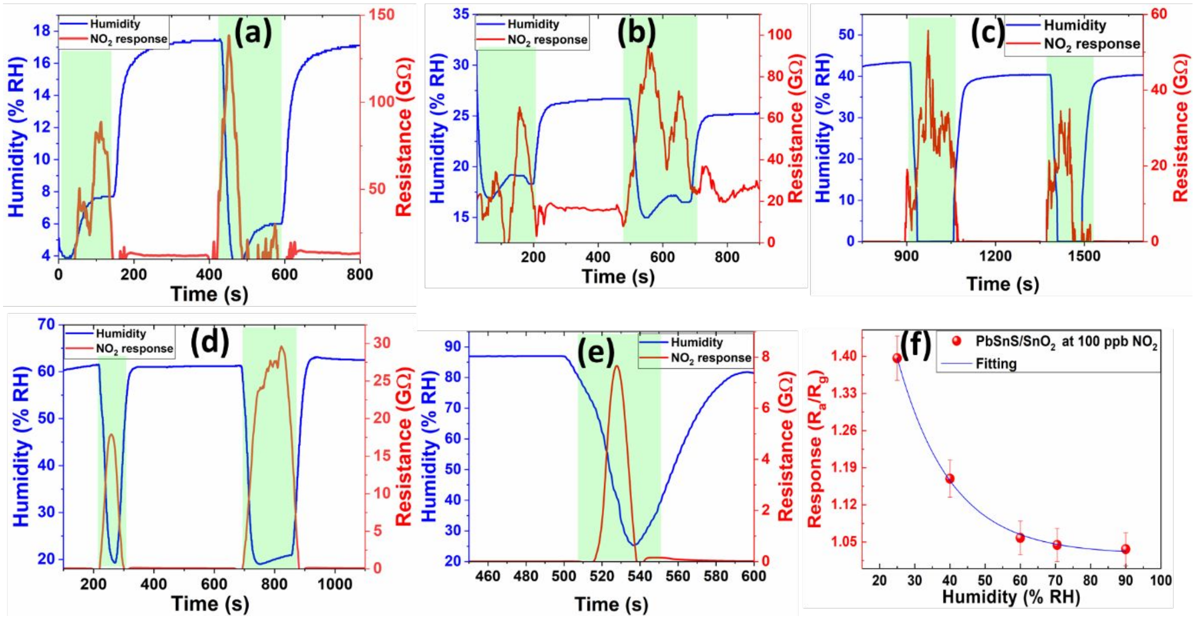

Fig. S6 (a-f) NO_2 sensing response at different levels of humidity
